# Acceptability, Engagement, and Exploratory Outcomes of an Emotional Well-being App: Mixed Methods Preliminary Evaluation and Descriptive Analysis

**DOI:** 10.2196/31064

**Published:** 2021-11-01

**Authors:** Amelia Eisenstadt, Shaun Liverpool, Athina-Marina Metaxa, Roberta Maria Ciuvat, Courtney Carlsson

**Affiliations:** 1 Evidence Based Practice Unit University College London and Anna Freud National Centre for Children and Families London United Kingdom; 2 Faculty of Health, Social Care and Medicine Edge Hill University Ormskirk United Kingdom; 3 Nuffield Department of Primary Care Health Sciences University of Oxford Oxford United Kingdom; 4 Division of Psychology and Language Sciences University College London London United Kingdom; 5 Paradym London United Kingdom

**Keywords:** smartphone, app, well-being, awareness, mental health, formative, mobile phone

## Abstract

**Background:**

There is growing evidence suggesting that the emotional well-being of the public has been negatively affected in the past year. Consequently, demand for well-being support has increased. Although there is substantial empirical support for mental health apps that target diagnosed conditions, there is less research on emotional well-being apps. Among existing well-being apps, few studies have been conducted on apps that are based on lived experience and those that seek to enhance users’ understanding of their emotional patterns. Thus, the acceptability of these novel apps requires further evaluation before upscaling.

**Objective:**

This evaluation aims to describe the acceptability, engagement, and preliminary outcomes of using an app (Paradym) designed to promote emotional well-being and positive mental health.

**Methods:**

This is a pre-post, mixed-methods, single-arm evaluation that is aggregated with digital analytics data. We anonymously collected real-world data on the demographics and well-being of the participants as well as the usability and acceptance of the app using validated questionnaires and open-ended questions. Participants tested the app for a minimum of 2 weeks before completing the follow-up measures. Google Analytics was used to record the level of app engagement. Chi-square and 2-tailed *t* tests were conducted to analyze quantitative data, and a thematic analysis approach was adopted for qualitative data.

**Results:**

A total of 115 participants completed baseline questionnaires, of which 79.1% (91/115) users downloaded the app. The sample was diverse in terms of ethnicity, including 43.4% (50/115) people who self-identified as belonging to minority ethnic groups. Most of the participants were female (78/115, 67.8%) and between the ages of 18 and 25 years (39/115, 33.9%). A total of 34 app users who completed questionnaires at baseline and follow-up provided valuable feedback to inform the future directions of Paradym. Favorable themes emerged describing the app’s content, functionality, and underlying principles. Although usability feedback varied across items, a considerable number of participants (22/34, 64%) found that the app was easy to use. Google Analytics revealed that at least 79% (27/34) of people used the app daily. On the basis of preliminary observations, app users experience increased mental well-being. Post hoc analyses indicated that the reduction in depression scores (*t*_33_=−2.16) and the increase in the well-being measures (*t*_33_=2.87) were statistically significant. No adverse events were reported during the follow-up period.

**Conclusions:**

The findings of this evaluation are encouraging and document positive preliminary evidence for the Paradym app.

## Introduction

### Background

Emotional well-being, as an important predictor of human health and longevity, has the potential to reduce the risk of physical and mental health disorders, is well established in the literature [1-4]. Research suggests that emotional well-being has declined in the general population in the past year [5-7]. Therefore, there is an increased need for accessible support services to meet this demand [6]. Evidence suggests that one mechanism for improving levels of emotional well-being is raising one’s emotional self-awareness [8]. Furthermore, apps have been identified as a promising mode of delivery based on their accessibility, scalability, and potential to provide anonymous services [9].

### Emotional Well-being and Emotional Self-awareness

Emotional well-being is defined as *a component of mental health that includes happiness, interest in life, and satisfaction* [10]. Researchers and policy makers have made the case for the promotion of emotional well-being to advance human health and reduce the costs associated with poor mental well-being and increased risk of mental health disorders [1,3]. Emotional awareness refers to the ability to recognize one’s emotions as they are being experienced and communicate them [11]. Low levels of emotional awareness have been associated with poor emotional well-being and an increased risk of mental health disorders, such as depression [8,12,13]. High levels of emotional awareness have been associated with a reduction in depressive symptoms and increased positive affect and emotional regulation [14,15]. There is also evidence suggesting that emotional awareness can enable flexibility of behavioral responses to difficult emotions and contribute to improved relationships with others [15,16].

### Emotional Well-being Apps

On the basis of the positive associations between emotional self-awareness and emotional well-being, a growing number of digital interventions have adopted this approach [9,17,18]. These apps offer a route to support users in improving their emotional well-being through their personal digital devices (eg, smartphones and tablets). Therefore, well-being apps can be used at any time, independent of location, with the added benefit that users can use the apps privately [9]. To this end, there has been a growing demand for and development of well-being apps that have accelerated in recent years [19,20]. A systematic review of 52 apps revealed that within the existing range of well-being apps, the apps have aimed to increase users’ management of emotions primarily through mindfulness, cognitive behavioral, and mood tracking approaches [21]. For instance, popular meditation and breathing apps, such as HeadSpace and Calm, have encouraged users to become aware of emotions and manage thoughts and emotions through daily meditation and breathing exercises [22-25].

Other apps have provided strategies from cognitive behavioral therapy, such as challenging negative thoughts, catastrophizing, and facilitating positive reappraisal [26-28]. Furthermore, some apps use mood tracking to enable users to identify emotions or moods and record and monitor these moods as an approach to emotional awareness [28-31]. In response to particular moods, some apps provide suggested activities to manage mood or emotional responses [30]. Although less frequently available, some well-being apps, such as MoodHacker, have been informed by principles of positive psychology that encourage users to identify their strengths and increase their mindful self-awareness [27].

Of the current range of well-being apps, it was noted that apps drew less frequently on lived experiences to explore well-being topics. Similarly, storytelling as a medium to convey psychoeducational concepts has been less studied in the literature [21]. The closest examples to this approach used fictional characters to guide users through the app [32] or real-life testimonials of other people who overcame difficult circumstances [33]. Further, to the best of our knowledge, there are no studied apps that encourage users to go beyond the exploration of their current emotional states, to inquire into their emotional patterns as a means of understanding their personality traits and tendencies over time [21]. Given the rapid development of apps, and relatively fewer studies on apps that use storytelling, lived experience, and emotional patterns, there appears to be a significant gap in this area that could benefit from further exploration. This is also important, given the need to explore wider techniques that could impact levels of engagement with digital interventions [21].

### The App Under Evaluation

The Paradym app was developed to support users in increasing their emotional awareness through learning about their emotional patterns to contribute to enhanced emotional well-being, increased self-awareness, and improved life satisfaction. The app uses storytelling as a starting point to introduce users to psychoeducational content that covers key areas of a person’s life (ie, love and relationships, body image, work and success, and identity). The app also incorporates concepts drawn from lived experience, which refers to the first-hand personal involvement of the user and the meaning that their past experience brings to current situations [34]**.** As a starting point, the app was designed for young adults aged 18 years and above. Before further development of the app and to ensure successful implementation, concepts of the Technology Acceptance Model were applied [35]. The model proposes that the assessment of perceived usefulness and perceived ease of use can determine whether users will engage with the new digital intervention. It is also recommended that user anxiety be monitored, as previous studies identified anxiety as an external variable in the technology acceptance model [36].

### The Process of Evaluation

The current evaluation was further guided by the Medical Research Council guidelines, which suggest addressing possible uncertainties identified during the development of new interventions [37]. Therefore, in this evaluation, we aimed to describe the acceptability, engagement, and preliminary outcomes of using an app (Paradym) designed to promote emotional well-being by adopting novel approaches. At this formative stage, the paper also aimed to record and report any negative consequences of using the app. To address these aims, the following key questions were addressed (Textbox 1).

**Key questions.**

**Engagement**

1. What are the characteristics of users accessing and using the app?
2. What are the users’ levels of engagement with the app?
3. Are there differences between completers and noncompleters?

**Acceptability**

1. What did users like about the app?
2. How usable or useful did users find the app?

**Preliminary outcomes**

1. Would users’ well-being increase after the intervention period?
2. Would users’ mental health symptoms (eg, depression) decrease after the intervention period?
3. Would participants experience severe or high levels of anxiety or other negative experiences during the intervention period?

**Feasibility**

1. Can users’ feedback be used to inform upgrades to the app?
2. Would it be feasible to conduct further evaluations of the app using current recruitment strategies and outcome measures?

## Methods

### Design

The current evaluation adopted a mixed-methods study design with a pre-post single-arm approach aggregate with digital analytics data. We adhered to the Consolidated Standards of Reporting Trials guidelines [38] where applicable, and registered the user testing protocol on the Open Science Framework a priori [39]. The only change in protocol was the decision to not collect data on anxiety at baseline. As the purpose of the app was not specifically designed to address mental health symptoms (eg, anxiety), to minimize the burden of completing lengthy questionnaires, we only collected data from the anxiety measure at follow-up. However, this was partly in line with our research question to explore whether the use of the app introduces high levels of anxiety.

### Participants

Real-world (ie, in-the-wild) data were collected from an international pool of potential users. User testing was advertised on Facebook campaigns and through social media over a 6-week period between May and June 2021. Anyone coming in contact with information about the user testing had the opportunity to participate in user testing. To be eligible for the user testing, participants needed to be over the age of 18 years and not have a diagnosable mental health condition.

### Procedure

Participants were enrolled in the user testing through a link that was provided on the web advertisement and on the Paradym website. The link provided access to web-based questionnaires for the baseline assessment and instructions on how to download the app from the Apple app store for iPhone users or Google Play for Android users. An automated email was sent to participants 2 weeks after completing the first battery of questionnaires for participants to complete the follow-up questionnaires. The questionnaires at time 2 included measures completed at time 1 and additional measures to capture the acceptability of the app and the users’ experience. Participants who completed questionnaires at both time points were entered into a free prize draw with the opportunity to win 1 of 3 US $25, US $50, and US $100 Amazon Gift Cards.

### Intervention Description

#### Overview

Paradym is an app aimed at supporting emotional well-being within the general population and targets adults over 18 years of age. It aims to provide users with support to develop greater emotional awareness and enhanced life satisfaction. Paradym was initially a crowdfunded project in the early stages of its development specifically to ensure that commercial interests were not taken into account in the early development of the intervention. The app provides a low-cost self-guided program with the aim of supporting users’ levels of emotional awareness and emotional well-being. Paradym is a standalone app, but it can also be used in combination with web-based group coaching sessions. However, for this evaluation, participants only accessed the standalone app.

Paradym was designed drawing on evidence-based strategies and designed in response to key topics that users identified as having such support as success, body, identity, love, and relationships. Clinical psychologists, coaches, and researchers were involved in the development of the content and the selection and review of psychoeducational content, suitable evidence-based strategies, and exercises (eg, journaling and recording personal notes).

Although an integrative theoretical approach underpins Paradym, key therapeutic theories such as acceptance and commitment therapy [40] and schema therapy [41] informed the lived experience and storytelling activities. Following consultation with users, further theory-informed strategies were applied to produce content that users indicated were important. Paradym comprises the following key strategies:

#### Digital Lessons

Psychoeducational digital lessons are structured into 5 pillars: aware, success, love, identity, and body. These topics were chosen based on the results of an end-user consultation conducted early in the development phase. Examples of psychoeducational content include an introduction to developing emotional awareness [42], supporting users with the identification and understanding of their emotions [43], and identifying and strengthening values [40].

Each digital lesson begins with an explanation of the psychoeducational concept based on lived experiences through personal storytelling. Storytelling is expected to help make the psychological content relatable, foster perspective taking, and support identification with the storyteller [44-46]. Storytelling has been found to boost engagement in both psychological interventions and apps [47]. Digital lessons are provided as chapters for reading, audio for listening, and video to watch speakers present the content to support different learning styles.

#### Emotional Patterns

At the end of each digital lesson, the user is asked to identify their own emotional pattern in relation to the app’s content, which draws on some concepts from schema therapy [41]. Its purpose is to enable the user to gain greater emotional awareness. To achieve this, the app introduces users to a range of emotional patterns and asks users to reflect on their own emotional patterns, and which pattern they perceive themselves to correspond to at the time of completing the lesson. Participants can then revisit specific emotional patterns and change them over time.

#### Reflections

Reflections are provided to the users via push notifications to prompt engagement with the app through a new daily reflection. Research shows that there are many benefits of reflection, such as supporting determination with tasks despite a stressful context [48], and that reflection may reduce rumination, which is linked with internalizing difficulties and interpersonal conflict [49].

Furthermore, notifications have been found to increase app engagement. Several studies have indicated that the use of mobile phone app interventions delivering psychological content must be combined with the active engagement of users [50,51].

### Data Collection Tools

#### Demographics

To understand the demographic profile of users accessing and using the interventions, participants were asked to provide anonymous information about their age, gender, ethnicity, and country of residence. Age was captured using age groupings of approximately 10 years (eg, 18-25 years, 26-35 years, 36-45 years). To allow inclusivity, participants were asked to enter their gender and ethnicity using free text. Participants also selected their geographic location (eg, city, state, country) from a global list. For analysis, ethnicity was dichotomized as White or minority ethnic groups. Age was recategorized to represent 45+ years, as there were fewer persons in each age category above 45 years. Location was then categorized as the United Kingdom, United States, and others to represent most responses.

#### Emotional Well-being and Mental Health Factors

##### Overview

The outcome measures used to assess well-being were the World Health Organization-Five Well-Being Index (WHO-5) [52], the Satisfaction With Life Scale (SWLS) [53], the Emotional Self-Awareness Scale-Revised (ESAS-R) [8], the Patient Health Questionnaire-9 (PHQ-9) [54], and the Generalized Anxiety Disorder-7 (GAD-7) [55]. WHO-5, SWLS, ESAS-R, and PHQ-9 were administered pre and post app use. The GAD-7 was administered post app use alongside the System Usability Scale (SUS) [56] and the semistructured acceptability questionnaire.

##### Well-being Measure

The WHO-5 allows a brief assessment (under 1 minute) of well-being over a 2-week period [52]. Individuals are asked to indicate for each of the 5 statements how they felt over the past 2 weeks using a 6-point Likert scale ranging from 0=*at no time* to 5=*all of the time*. The WHO-5 is derived from a 28-item version based on items from the Zung scales for depression, distress, and anxiety, as well as from the General Health Questionnaire and the Psychological General Well-Being Scale [57]. The WHO-5 has been validated as a measure of depression in both adolescents and older adults, with high measurement invariance [58]. A high score indicated a high level of well-being.

##### Satisfaction With Life Measure

The SWLS [53] is a short 5-item scale designed to measure the global cognitive assessment of satisfaction with one’s life. The estimated time for completion of SWLS has been reported to be approximately 1 minute. The SWLS has been shown to have very high construct validity, with Cronbach α=.85-.87 [59] and moderately high reliability (Cronbach α=.78) [60]. A high score obtained from the SWLS indicates a high level of life satisfaction.

##### Emotional Self-awareness Measure

The ESAS-R [8] is a 30-item scale, and all items are rated on a 5-point Likert scale ranging from 0 to 5 (0=never, 1=very little, 2=sometimes, 3=often, 4=a lot). The subscales ranged from 0 to 20. The total scale ranged from 0 to 132. Subscales included recognition, identification, communication, and contextualization. The ESAS-R has been shown to have high validity and reliability (Cronbach α=0.83-0.90) [30]. A high score on this measure indicates a high level of emotional self-awareness.

##### Depression Measure

The Patient Health Questionnaire (PHQ-9) is a depression scale that scores each of the 9 *Diagnostic and Statistical Manual of Mental Disorders, Fourth Edition* criteria as 0 (not at all) to 3 (nearly every day). The PHQ-9 has been validated for use in primary care [45]. It is not a screening tool for depression, but can monitor the severity of symptoms and response to treatment. Scores over 10 have good sensitivity (88%) and specificity (88%) for the diagnosis of major depression by interview. It has high internal reliability, and Cronbach α=.89 [61]. The construct validity of the PHQ-9 is also high for community and clinical samples [62]. A high score obtained from the PHQ-9 is indicative of a severe level of depression.

##### Anxiety Measure

The GAD-7 is a 7-item, brief clinical measure that assesses the presence and severity of generalized anxiety disorder. The self-report scale asks how often during the last 2 weeks individuals experienced symptoms of generalized anxiety disorder. Total scores range from 0 to 21, with cutoff scores of 5, 10, and 15 indicating mild, moderate, and severe anxiety, respectively. Increasing scores on the GAD-7 are strongly associated with greater functional impairment in real-world settings. Sample items are rated from 0 (not at all) to 3 (nearly every day). Scores over 10 have good sensitivity (89%) and specificity (82%) for diagnosis of generalized anxiety disorder by interview, and the scale has high internal reliability, as suggested by a Cronbach α=.92 [55]. The scale has been widely used and considered a valid and reliable screening tool in previous research, presenting good reliability, factorial, and concurrent validity [63]. A high GAD-7 score is an indicator of severe anxiety.

#### Acceptability and Usability

##### System Usability Scale

The SUS is a 10-item scale with all items measured on a 5-point Likert scale ranging from 1 to 5 (1=strongly disagree and 5=strongly agree) with total scores ranging from 0 to 100. This widely used scale in technology provides an overview of the subjective assessments of usability [56]. The SUS has been shown to have excellent construct validity and reliability (0.81-0.94) [64,65]. A high score on the SUS indicates a high level of usability, with a score above 68 considered above average.

##### Semistructured Questions

In addition to the SUS, we aimed to capture information about the acceptability of the app by identifying facilitators and barriers to using the app. For the open-ended questions, the following questions were adapted from a study by Kern et al [66] to fit the current evaluation:

1. Why would you use (Paradym)?
2. Why would you not use (Paradym)?
3. Explain why (or why not) you would prefer to use (Paradym) to seeing a mental health professional?

##### Adverse Events

To capture any side effects of using the app, participants were provided with the email address for the research team and requested to contact a member of the research team if they experienced any distress or discomfort or experienced any other issue because of their use. Adverse events (ie, side effects) were identified and recorded as any untoward medical or clinical occurrence, which did not necessarily have a causal relationship with the intervention. Any adverse events arising during the evaluation period were assessed for severity, causality, seriousness, and expectedness (ie, relating to the information provided by the app).

### Data Analysis

Descriptive statistics were calculated for participant characteristics at baseline (preintervention), and Google Analytics estimates were used to report app engagement. The main focus was on descriptive data to address the aims of the evaluation. However, some exploratory significance tests were conducted on within-group mean differences at the 2 time points (ie, baseline and follow-up) on mental health and well-being measures. Paired samples, 2-tailed *t* tests were conducted on continuous data, and Chi-square tests were used for categorical variables in SPSS [67]. Qualitative data from the semistructured open-ended questions were captured in a questionnaire format, coded in categories, and analyzed using the steps outlined for thematic analysis [68]. The data were coded line by line and then clustered into provisional themes. Each candidate theme was reviewed by going back to the coded data to check whether the meaning was accurate. Next, the uncoded datasets were revised to ensure that the data were relevant to the research question. Finally, the provisional theme titles and the codes that they contained were checked to confirm that they remained relevant and accurate. Any discrepancies that arose were discussed at team meetings and only included after a consensus was reached.

### Ethical Considerations

We were guided by the research ethical principles for ensuring the rights, safety, dignity, and well-being of the participants in this evaluation were protected [69]. All data were collected remotely from a nonclinical population, and participants voluntarily provided informed consent for their anonymized deidentifiable data to be used for research purposes. The research team’s email address was available for participants to contact us if they experienced any discomfort because of using the app. The researchers were prepared to signpost users to relevant well-being support and resources, if required. Owing to the formative nature of the evaluation, findings were not expected to be generalizable or transferable in any way. We also consulted other researchers affiliated with academic institutions and practitioners known to the study team who reviewed the evaluation protocol and provided oversight with respect to research ethical guidance. In light of the above, this evaluation was viewed as user testing or service evaluation and therefore was exempt from formal ethical approval by the Health Research Authority (Multimedia Appendix 1).

No formal ethical approval was required for similar acceptability studies [30,70,71]. As explained by Ahtinen et al [72], ethics committee approval was not acquired, as the study was deemed to involve minimal risk and the focus was on studying mainly user experiences. In addition, the present evaluation does not involve a clinical population, like that of Ly et al [73]: “Since this pilot trial involved a non-clinical population, it was considered exempt from registration in a public trials registry.”

## Results

### Overview

Questions about engagement, acceptability, and preliminary outcomes (1-8) are addressed in the results section, and questions on feasibility (9,10) are addressed in the *Future Directions* subsection of the *Discussion* section.

### Descriptive Data

**Figure 1:**
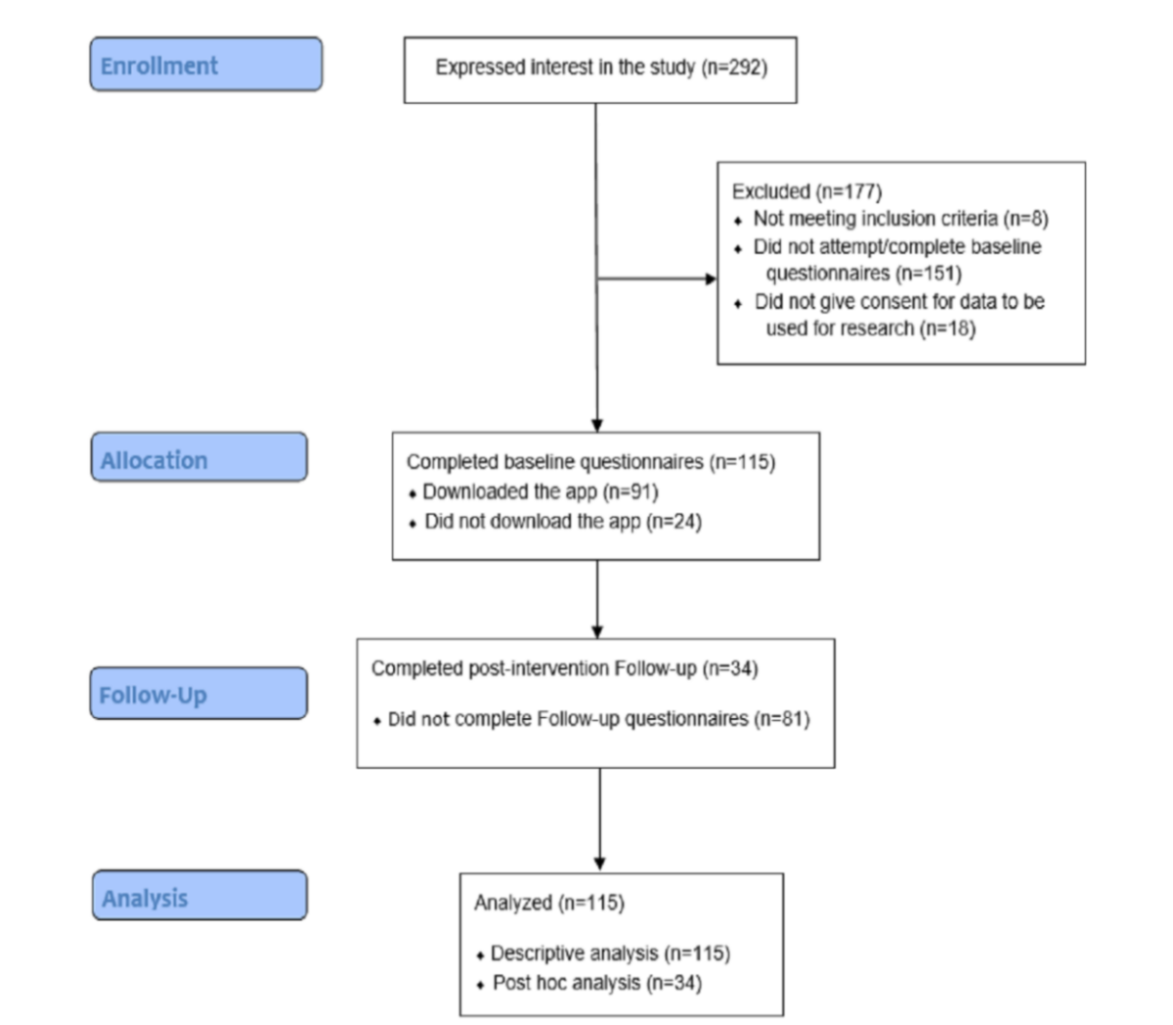
Flowchart of participants' progress through the evaluation period.

During the 2 weeks, the user testing was advertised, and 292 participants expressed interest as part of the preliminary evaluation. One hundred fifty-one individuals either did not attempt or did not complete the baseline questionnaires and therefore did not reach the relevant page to be able to download the app. However, data from 18 participants were not considered, as they did not provide consent to process their information for research purposes. Data from 8 participants were excluded as they indicated that they were not eligible for the evaluation based on mental health diagnoses. Consequently, data from 115 participants (ie, completing baseline questionnaires and providing consent) were included in the evaluation. Of these, 91 participants downloaded the app, and 34 of the participants completed questionnaires at both baseline and follow-up and were included in the final analysis. Figure 1 depicts the flow of participants throughout the evaluation period.

### Participant Characteristics

At baseline (n=115), the participants were a self-identified diverse sample with 43.4% (50/115) belonging to minority ethnic groups, with most being female (78/115, 67.8%). Most of the samples (80/115, 69.5%) were from different cities across the United States. Participants’ ages ranged from 18 to 65+ years, with the mode being 18 to 25 years. Table 1 provides further details of the sample. As for mental health and well-being characteristics, the participants were generally *dissatisfied* with their life (mean 20.16, SD 6.24) and had *moderate* symptoms of low mood or depression (PHQ-9; mean 10.35, SD 6.65). Participants also reported *average* scores on emotional self-awareness (ESAS; mean 56.95, SD 13.94) and general well-being (WHO-5; mean 17.22, SD 4.96).

**Table 1:** Demographic information for the participants at baseline and follow-up.

Characteristics			Participant, n (%)		
			Baseline (n=115)		Follow-up (n=34)
**Gender**					
	Male	29 (25.2)		9 (26.5)	
	Female	79 (68.6)		23 (67.6)	
	Transgender	2 (1.7)		0 (0)	
	Other or prefer not to answer	7 (6)		2 (5.9)	
**Age (years)**					
	18-25	39 (33.9)		13 (38.2)	
	26-30	21 (18.2)		3 (8.8)	
	31-35	15 (13)		0 (0)	
	36-40	15 (13)		5 (14.7)	
	41-45	7 (6)		4 (11.8)	
	46-50	4 (3.4)		0 (0)	
	51+	15 (13)		9 (26.5)	
**Ethnicity or race**					
	White	66 (57.3)		24 (70.6)	
	Black	12 (10.4)		2 (5.9)	
	Hispanic	4 (3.4)		0 (0)	
	Asian	20 (17.3)		6 (17.6)	
	Indian	1 (0.8)		0 (0)	
	Asian Americans and Pacific Islanders	1 (0.8)		1 (2.9)	
	Jewish	2 (1.7)		0 (0)	
	Mixed	10 (8.6)		1 (2.9)	
**Country**					
	United States	80 (69.5)		23 (67.6)	
	United Kingdom	32 (27.8)		10 (29.4)	
	Other	1 (0.8)		1 (2.9)	
	Did not answer	3 (2.6)		0 (0)	

### Engagement With the Intervention

App use data were made anonymous to comply with the General Data Protection Regulation and research ethical guidelines. Ninety-one users downloaded the app; therefore, it was not possible to separate the data of users who used the app only and those that used the app and completed the final battery of measures. During the evaluation period, all users logged into the app at least once. The average use across all participants during the evaluation period was 23.53 minutes. The reflections received 215 views. Users returned to the app an average of 4.5 times with each session lasting an average of 5.22 minutes. Overall, the Daily Active Use Monthly Active Use ratio (ie, the proportion of users that engage with the app in a single day, calculated by dividing the number of daily users by the number of monthly users) was 80% according to Google Analytics.

### Attrition or Dropout

In terms of attrition, 70.4% (81/115) users did not complete follow-up questionnaires. The remaining 29.5% (34/1115) participants completed the questionnaires at the 2 required time points. Overall, there were no significant differences in demographic or mental health or well-being characteristics. However, based on descriptive data, participants who completed the evaluation had slightly lower mean scores at baseline on all outcome measures including SWLS (18.94 vs 20.67), WHO-5 (16.18 vs 17.65), ESAS-R (55.95 vs 57.37) and PHQ-9 (10.12 vs 10.45).

### Acceptability

Thematic analysis of the qualitative data led to the development of 3 key themes. Participants provided favorable feedback describing acceptance of *the app’s content*, *the app’s functionality*, and *the app’s underpinning principle*. Owing to the interaction of these themes, users’ feedback indicated that there is an element of app versatility, with users describing the use of the app at different times of the day. Some described it as part of their morning routine or used the Paradym app in conjunction with other well-being and health apps.

### The App’s Content

In this first theme, users commented on the content of the app, expressing positive evaluations of the app’s chapters, reflections, and exercises, and reported that listening to or reading the content helped them with their understanding of themselves and their mental health. For example, one user expressed, “[Because of the app, I am] studying myself more closely which has benefited my mental health” (Female, 51+ years).

Other users recognized the value of Paradym as a form of additional support separate from that of a professional. They said that Paradym could assist with self-help, whereas their mental health professionals could help with the application of the in-app content. For example, one user expressed, “Paradym allows me to individually learn and apply it onto myself while training myself also. Seeing a mental health professional could allow them to help me directly” (Female, 18-25 years).

### The App’s Functionality

In this second theme, users reported that the combination of videos and text as aspects of the app’s functionality was helpful when working through the exercises as a way of building knowledge. For example, one user said, “The videos were actually pretty engaging when I got around to watching them, and I did feel like I gleaned some insights and self-awareness from that” (Gender not reported, 18-25 years).

Others reported valuing the app as a chance to reflect and understand emotions and found the daily reflection notifications useful: “[A chance to] reflect on my emotions and life. Works best with the daily prompts” (Female, 18-25 years).

### The App’s Underpinning Principle

In the third theme, users commented on the app’s underlying principle. Participants reported benefits from building increased awareness of their emotional and behavioral patterns, moods, and having an opportunity to explore and record them. For example, one participant said, “[The app allowed...] building consistency and having a place to collect my thoughts about my mood and behavioral patterns” (Female, 26-30 years).

Similarly, another user reported that developing self-awareness was important and linked to their personal aspirations. For example, one participant reported, “[I would use Paradym...] to discover bits about myself and become the person I knew I was capable of being” (Male, 41-45 years).

### Usability Analysis

The mean usability score was almost 60 (mean 59.77, SD 23.65) out of a possible 100. Participant responses varied across the items on the SUS. On the learnability subscale, over 91% (31/34) of the sample were neutral or disagreed that they would need help to use the app (Item 4), and over 82% (28/34) were neutral or agreed that they could learn to use the app quickly (Item 7). In the same vein, 74% (25/34) of users reported feeling confident enough to use the app (Item 9). Table 2 provides further details on the participants’ usability experience.

**Table 2:** Systems usability scale self-report of items post intervention.

Number	Item	Agree, n (%)	Neutral, n (%)	Disagree, n (%)	Missing data, n (%)
1	I think that I would like to use Paradym frequently	14 (41)	9 (26)	11 (33)	0 (0)
2	I found Paradym unnecessarily complex	12 (35)	6 (18)	16 (47)	0 (0)
3	I thought Paradym was easy to use	14 (41)	11 (33)	9 (26)	0 (0)
4	I think that I would need the support of a technical person to be able to use Paradym	3 (9)	6 (18)	25 (73)	0 (0)
5	I found the various functions in this app were well integrated	15 (42)	12 (35)	7 (23)	0 (0)
6	I thought there was too much inconsistency in Paradym	7 (20.6)	9 (26.5)	17 (50)	1 (2.94)
7	I would imagine that most people would learn to use Paradym very quickly	22 (64.7)	6 (17.6)	5 (14.7)	1 (2.94)
8	I found Paradym very cumbersome to use	12 (35.3)	7 (20.6)	14 (41.2)	1 (2.94)
9	I felt very confident using Paradym	17 (50)	8 (23.5)	9 (26.5)	0 (0)
10	I needed to learn a lot of things before I could get going with Paradym	7 (20.6)	8 (23.5)	17 (50)	2 (5.88)

### Adverse Events

Regarding possible side effects or adverse events, no reports from the participants were received during the evaluation period. Therefore, the research team was unaware of any harmful outcomes that could result from using the app.

### Preliminary Efficacy

On the basis of the available data from the participants who completed the evaluation, a descriptive analysis showed that mean scores slightly improved on the SWLS (+1.56), the WHO-5 (+2.03), and the ESAS (+1.75). However, the increase was statistically significant only for the WHO-5 score (*t*_33_=2.87). In addition, participants’ depression scores also decreased over time (−1.56) and were found to be statistically significant (*t*_33_=−2.16). Participants’ mean anxiety levels were within the mild category of about 8.24 (SD 6.59) during the intervention period. Table 3 provides further descriptions of the sample (n=34) included in the final analyses.

**Table 3:** Descriptive statistics for each outcome measure at baseline and follow-up.

Outcome measure	Baseline, mean (SD)	Follow-up, mean (SD)	Coefficient (95% CI)	Cohen *d*	*P* value
SWLS^a^	18.94 (5.76)	20.50 (7.03)	0.73 (−0.11 to 0.85)	0.37	.07
WHO-5^b^	16.18 (4.70)	18.21 (4.94)	0.64 (0.02 to 0.99)	0.51	.007
ESAS-R^c^	55.95 (12.50)	57.70 (14.27)	0.50 (−0.34 to 0.62)	0.14	.57
PHQ-9^d^	10.12 (6.25)	8.56 (6.35)	0.78 (−0.85 to 0.11)	−0.38	.04
GAD-7^e^	—^f^	8.24 (6.59)	—	—	—

^a^SWLS: Satisfaction with Life Scale.

^b^WHO-5: World Health Organization-Five Well-Being Index.

^c^ESAS-R: Emotional Self-Awareness Scale-Revised.

^d^PHQ-9: Patient Health Questionnaire-9.

^e^GAD-7: Generalized Anxiety Disorder-7.

^f^Not available.

## Discussion

### Principal Findings

The aim of this user testing and initial service evaluation was to describe the acceptability, engagement, and preliminary outcomes of using a new version of a well-being app. The findings indicated that users who downloaded the app (n=91) generally accessed and used the app during the intervention period for an average of 5.22 minutes with an average of 4.5 sessions per user over the 2-week period. A fair number of participants who completed the post assessment (10/34, 29.4%) engaged with the app content in all 5 pillars. Despite the observed level of engagement (mean 23.53 minutes), there was high attrition (70.4%) during the evaluation period. Although there were no significant differences between the users who completed the evaluation and those who dropped out, the mean scores for mental health and well-being measures were higher in those who dropped out. It is possible that people who experienced more psychosocial difficulties found it difficult to engage with the app or to respond to the outcome measures [74]. Mental health apps generally have high dropout rates, and some studies have found that once users learn a skill or knowledge from a particular app, they stop using it [75]. It is also conceivable that, for users with high life satisfaction, they may have had less need for the knowledge provided in the app or felt they had obtained what they needed before the end of the 2-week period.

Participants who completed the evaluation provided favorable feedback in terms of the app’s content, functionality, and underlying principles. Although usability feedback varied across items of the SUS, a high percentage of participants found that it was easy to use the app. This can be attributed to evidence suggesting the importance of specific features that could influence users’ experiences. For example, younger digital users are more likely to engage with interventions that have features such as videos, limited text, ability to personalize, ability to connect with others, and options to receive text message reminders [76]. Moreover, the promotion of increased self-awareness is becoming popular among young people in their early twenties [77]. Therefore, as emotional awareness is one of the main purposes of Paradym, and a fair amount of content is presented via videos or audio, users could have responded particularly favorably. Similarly, Paradym includes customization features, exercises, and text materials. Contrary to Liverpool et al [76], users in this evaluation responded favorably to text-based content (as per the digital lessons). Further research is needed to determine whether specific features may have more or less impact on use among various age groups.

In addition, based on descriptive data, all mental health and well-being scores improved. The change in well-being (WHO-5) and depression (PHQ-9) were statistically significant, indicating that the use of the app could potentially improve some symptoms related to poor well-being. However, as this was not a controlled study, it is not possible to make any causal claims or explicitly attribute the findings to the use of the app. Nonetheless, this is the first study to evaluate Paradym; therefore, these preliminary findings can be viewed as positive and warrant further investigation. The other outcome measures did not yield statistically significant results. This could be because of the small sample size (n=34) or other methodological issues, such as the 2-week intervention period. It could be that factors associated with life satisfaction and emotional awareness require longer use periods. It was also noted that anxiety levels of the participants were mild during the intervention period, which at a minimum could indicate that the Paradym app did not induce any unnecessarily high levels of anxiety.

### Comparison to Other Findings

In terms of the demographic profile of the sample, the evaluation captured data from a diverse sample (50/115, 43.5%, belong to minority ethnic groups). This is generally uncommon in mental health app studies [22] and therefore provides valuable insights from underserved and underrepresented populations. Although we hoped to collect data from a larger sample, the total sample size and high attrition observed in this evaluation appear to be common when evaluating digital interventions [78]. Despite these challenges, the findings are in line with the existing literature, indicating the potential usefulness of apps to support positive mental health and well-being. Our findings suggest a significant increase in well-being, which has also been observed in similar studies [22,24]. A decrease in depression symptoms has also been reported in other studies that evaluated mental health app use [31,79-81]. Similar themes and statistics have also been reported for user engagement and acceptance [82,83]. However, in terms of study design, this evaluation obtained both quantitative and qualitative data, including objective engagement data, to fully capture users’ feedback about the app. This evaluation also demonstrated that it was possible to conduct an app evaluation within a 6-week period, whereas previous studies reported slightly longer evaluation periods of 8 weeks [84] or more [85]. When compared with recent systematic reviews and meta analyses, the effect sizes in our evaluation fell within the previously observed range (eg, 0.10-0.57) for depression (k=12 [86]; k=18 [87]). Furthermore, for well-being, the effect size in this evaluation (0.51) was above the previously observed range (k=5; effect size range: 0.14-0.45) [88].

In terms of the app itself, based on available descriptions for similar apps [89], Paradym, to the best of our knowledge, may be one of the first apps that focuses on improving emotional awareness and emotional well-being for the general population through the medium of identifying emotional patterns, and integrates many psychological theories. The module-based content is similar to that of other apps [90-92], but the focus on users identifying and selecting a range of emotional patterns to build an overall profile appears to be unique to Paradym. This is an important finding, as preliminary feedback suggests that some users responded positively to this feature.

### Strengths and Limitations

The main strength of this evaluation is its ability to collect and analyze data from a diverse sample of app users. At this preliminary stage of the app’s evaluation, the findings already suggest some evidence of promise. These include significant impacts on mental health and well-being and no reports of adverse events, in addition to positive feedback on use and engagement. Another strength is its ability to recruit an acceptable sample size and carry out the evaluation within a 6-week period. This could indicate a need on behalf of the users and demonstrate to other app developers and researchers that it is feasible to conduct ongoing evaluations during rapid prototyping phases.

Despite its notable strengths, this evaluation also has some limitations. Obvious limitations include the single-arm pre-post evaluation design, which limits the ability to perform comparative analyses. The moderate sample size (n=34) has limited potential to carry out subgroup analyses (eg, males). Studies with larger sample sizes and subgroup analyses could provide valuable information for future versions of the app. The high attrition rate (70.4%), which is in line with results reported by other studies, is a point of concern, and future evaluations of the app would take this into account during the recruitment period. This limitation could be attributed to data collection via a link outside of the app platform because the engagement data showed higher numbers of people (n=91) continuing to use the app without completing the outcome measures. Owing to the anonymous nature of the evaluation, it was not possible to make comparisons between participants who downloaded the app and those who did not. Another limitation was the 2-week intervention period. Although this can be viewed as a strength for this preliminary evaluation, there may be benefits, such as observations about sustained use and further impact on well-being, if a longer app use period was included. Another limitation lies in the brief responses provided by the users for the semistructured questions. This limited the amount of data available to provide deeper insights into thematic analysis. These limitations and other key learnings will be considered in future evaluations of the app.

### Future Directions

#### App Upgrades

Participants provided valuable feedback that had already been absorbed to inform upgrades to the app. On the basis of these findings, the following upgrades will be prioritized before further evaluations of the app are conducted. First, a proportion of participants expressed that they would like the app to be more interactive. A guided process has been added as an adjunct to the app so that users can join weekly sessions as part of a group to discuss sections of the app and reflect on concepts of self-awareness with an assigned coach. Second, the feedback indicated that some users forgot about the app and needed reminders. More in-app notifications have been included in newer versions of the app and are expected to further improve the levels of engagement [93,94]. Third, based on recent findings for increasing engagement [24,95] and the positive perception of the app’s content and functionality, features such as personalized check-ins will be considered for inclusion in the app to further enhance the users’ experience.

#### Research Implications

This evaluation provides a strong foundation for future evaluations of apps. The current recruitment, intervention, and data collection period (for a total of 6 weeks) may be too ambitious to gather a large enough sample size to meaningfully test statistical significance on multiple variables. On the basis of our findings and the experience of carrying out this evaluation, 12 weeks may be more feasible, allowing at least 1 month for each stage of the study. In this way, participants will have a longer period to engage with the app and therefore complete all modules within the app. The outcome measures used appear to be appropriate, with almost 100% completion (for n=34 users) on all items. Future evaluations of the app could maintain the current data collection tools and include the anxiety measure (ie, GAD-7) at baseline to ensure that it accurately captures any direct influence of the app on participants’ levels of anxiety. Therefore, this approach may go beyond the research question addressed in this evaluation and provide data on any relationship the app may have with lowering anxiety. This may prove to be valuable information that could substantiate the findings highlighted in the relationship between the app and depression scores. It may be just as useful to enhance the evaluation by adding a comparative group such as a blended intervention group and randomizing participants to ensure that our findings do not occur by chance. In future evaluations, it may be just as important for us to collect qualitative data from participants who drop out to fully capture if their decision to discontinue is based on app use or lack of time to complete outcome measures, as suggested by other researchers [96].

#### Practical Implications

Given the range of well-being apps, and the preliminary positive implications of this evaluation, improving well-being through regular use of an emotional well-being app that focuses on emotional awareness and knowledge of emotional patterns, could have potential promise as a low-cost approach to increase well-being and related outcomes, such as life satisfaction. Sustaining user engagement in apps over time is not straightforward. The results of this evaluation are preliminary because of the moderate sample size across data collection points; however, there may be benefits to apps that draw on lived experience and storytelling, are multimodal, and promote increased emotional awareness and understanding of emotional patterns. This evaluation recruited a higher-than-average number of participants from minority ethnic backgrounds, and further apps and research initiatives ought to appeal to nonmajority app users.

### Conclusions

This evaluation further highlighted the value of conducting formative research on mental health and well-being apps. For example, the findings suggest that users can provide valid feedback that can be used to inform future app upgrades. Users generally engaged with the app and provided favorable feedback regarding the app’s content, functionality, and underlying principles. Notably, participants in this evaluation experienced significantly lower levels of depression scores after the intervention period, as well as increased well-being. The findings of this evaluation are encouraging and show positive preliminary evidence that suggests scope for further research with underrepresented groups, such as ethnic minority populations.

## Appendix

Multimedia Appendix 1 Ethical review decision tool developed by the Medical Research Council.

## Abbreviations

ESAS-R: Emotional Self-Awareness Scale-Revised
GAD-7: Generalized Anxiety Disorder-7
PHQ-9: Patient Health Questionnaire-9
SUS: System Usability Scale
SWLS: Satisfaction With Life Scale
WHO-5: World Health Organization-Five Well-Being Index
